# Noninvasive Immuno-PET Imaging of CD8^+^ T Cell Behavior in Influenza A Virus-Infected Mice

**DOI:** 10.3389/fimmu.2021.777739

**Published:** 2021-11-03

**Authors:** Paul W. Rothlauf, Zeyang Li, Novalia Pishesha, Yushu Joy Xie, Andrew W. Woodham, Djenet Bousbaine, Stephen C. Kolifrath, Vincent L. Verschoor, Hidde L. Ploegh

**Affiliations:** ^1^ Program in Virology, Harvard Medical School, Boston, MA, United States; ^2^ Department of Molecular Microbiology, Washington University School of Medicine, St. Louis, MO, United States; ^3^ Program in Cellular and Molecular Medicine, Boston Children’s Hospital, Boston, MA, United States; ^4^ Society of Fellows, Harvard University, Cambridge, MA, United States; ^5^ Klarman Cell Observatory, Broad Institute of MIT and Harvard, Cambridge, MA, United States; ^6^ Department of Immunology and Infectious Diseases, Harvard School of Public Health, Boston, MA, United States; ^7^ Department of Pediatrics, Harvard Medical School, Boston, MA, United States; ^8^ Leiden Academic Centre for Drug Research, Leiden University, Leiden, Netherlands

**Keywords:** immuno-PET, influenza A virus, CD8, T cells, imaging

## Abstract

Immuno-positron emission tomography (immuno-PET) is a noninvasive imaging method that enables tracking of immune cells in living animals. We used a nanobody that recognizes mouse CD8α and labeled it with ^89^Zr to image mouse CD8^+^ T cells in the course of an infection with influenza A virus (IAV). The CD8^+^ signal showed a strong increase in the mediastinal lymph node (MLN) and thymus as early as 4 days post-infection (dpi), and as early as 6 dpi in the lungs. Over the course of the infection, CD8^+^ T cells were at first distributed diffusely throughout the lungs and then accumulated more selectively in specific regions of the lungs. These distributions correlated with morbidity as mice reached the peak of weight loss over this interval. CD8^+^ T cells obtained from control or IAV-infected mice showed a difference in their distribution and migration when comparing their fate upon labeling *ex vivo* with ^89^Zr-labeled anti-CD8α nanobody and transfer into infected versus control animals. CD8^+^ T cells from infected mice, upon transfer, appear to be trained to persist in the lungs, even of uninfected mice. Immuno-PET imaging thus allows noninvasive, dynamic monitoring of the immune response to infectious agents in living animals.

## Introduction

Influenza remains a serious disease, with an estimated 1 billion infections per year, some 290,000–650,000 of which are fatal (1). Influenza A virus (IAV), a segmented, negative-sense RNA virus in the family *Orthomyxoviridae*, causes seasonal epidemics. IAV can also cause pandemics, during which individuals have limited cross-protective immunity as a result of the virus’s ability to reassort its segments with heterologous influenza strains (2, 3). Despite the development of antivirals and vaccines, influenza virus-associated deaths remain a major concern. A better understanding of the clinical course of infection and the host’s immunological response to the virus is crucial to improve vaccines and therapeutics against influenza viruses.

IAV, along with the related influenza B virus, causes a range of clinical manifestations, from mild self-limiting respiratory tract infections, to progressive and sometimes lethal pneumonia (4). Both the upper and lower respiratory tracts are sites of viral replication, with nascent virions spreading amongst epithelial cells of the upper respiratory tract and trachea in most non-fatal cases. Lower respiratory involvement and pneumonia, caused by the virus and/or secondary bacterial infections, are often seen in fatal cases (5).

Upon detecting the presence of IAV infection, the host immune system clears the viral infection using both innate and adaptive immune components (6). The rapid induction of innate defenses is critical for early protection and limits viral spread to host tissues, while adaptive immunity develops. Early stages of innate immunity include the rapid production of type I interferons (IFNs), cytokines, and chemokines through activation of pattern recognition receptors, which in turn attracts inflammatory cells to the airways to help clear the infection and prepare the host for the adaptive immune response (7). The adaptive immune response is activated when lung-resident dendritic cells sense cytokines and acquire debris from infected, dying cells, which induces their migration from the inflamed lungs to the lung-draining lymph nodes (DLNs). There, they display newly acquired viral antigen(s), processed to yield peptide-MHC complexes (8). Naïve T cells in the DLNs recognize these complexes, expand clonally, and then differentiate into IAV antigen-specific CD8^+^ and CD4^+^ effector T cells (9). Secondary lymphoid organs, such as DLNs and the spleen, provide the spatial organization and appropriate chemokine environment to prime the antiviral immune response by bringing together Th1 CD4^+^ and naïve CD8^+^ cells to generate influenza-specific CD8^+^ cytotoxic T lymphocytes (CTLs). Virus-specific T lymphocytes then migrate to the site of infection in response to cytokines and chemokines, and B cells are primed to secrete neutralizing antibodies (10).

The virus itself causes extensive damage to and desquamation of the airway epithelium, but effector T cells also contribute to tissue damage directly by secreting granzymes and perforin, as well as by inducing FasL- and TRAIL-mediated apoptotic pathways (5, 11). CTL-induced tissue damage, however, is thought to be more extensive as a consequence of indirect mechanisms, such as by secretion of pro-inflammatory molecules that recruit other players to the site of infection, including IFNγ, macrophage inflammatory protein-1α, and CTL-derived tumor necrosis factor-α (12–18). Observing the spatiotemporal behavior of CTLs during IAV infection, ideally longitudinally and noninvasively, is essential to better understand the immunopathology induced by these cells and to eventually prevent collateral damage inflicted by T cells.

One technique used to visualize and track cells *in vivo* is positron emission tomography (PET), which is used to identify tumors in humans. PET scanning relies on detection of a radiotracer, such as ^18^F-2-fluorodeoxyglucose, which is absorbed by tumor cells with high metabolic activity. Immuno-PET is a technique that uses radiolabeled antibodies, or antibody fragments, that target disease- or cell-specific antigens to track the distribution of that antigen (19, 20). We have used immuno-PET, more specifically using single domain antibody fragments (VHHs or nanobodies) derived from alpaca heavy chain-only antibodies, to track immune cell distribution and infiltration in graft-versus-host disease, as well as in models of cancer and the immune response to cancer therapies (21–23).

We used radiolabeled nanobodies to study the kinetics of CD8^+^ T cell recruitment to the site of infection, using mouse-adapted IAV. We also identified the localization of CD8^+^ T cells from IAV-infected and control mice upon transfer to assess cell-autonomous migratory behaviors. By transferring labeled CD8^+^ T cells from IAV-infected mice into either healthy or IAV-infected recipients, we examined whether the inflamed environment of the infected lungs affects tissue-specific trafficking of both naïve and antigen-specific T cells. Prior studies have relied mostly on invasive techniques, such as tissue harvest, followed by flow cytometry to enumerate CD8^+^ T cells in the organ of interest. While this provides insight into the local distribution of CD8^+^ T cells, it does not provide information on changes in distribution or migration of CD8^+^ T cells to peripheral tissues in the course of infection. We show that it is possible to noninvasively monitor the total population of CD8^+^ T cells over the course of an IAV infection at millimeter spatial resolution. This approach provides a new means to track a CD8^+^ T cell response noninvasively in a living animal.

## Materials and Methods

### Synthesis of Peptide Probes for Sortase Reactions

Peptide GGGCGGSK(azide) with a free N-terminus and C-terminal amide was synthesized following standard solid phase synthesis protocols (24). All Fmoc amino acids were purchased from Chempep, Inc. Fmoc-Lys(azide)-OH was used as a building block to provide the bioorthogonal handle. Peptides were purified by reverse phase HPLC. Their identity was confirmed by LC-MS prior to maleimido-DFO coupling to the cysteine thiol. Peptides were further purified by reverse phase HPLC and their identity was confirmed again by LC-MS.

### C-Terminal Sortagging and PEGylation

Ca^2+^-independent heptamutant sortase A derived from *S. aureus* (10 μM final concentration, 10x stock in 50 mM Tris, pH 7.4, 150 mM NaCl) and probe (1 mM final concentration, 50x stock) were added to VHH-X118 (200 μM final concentration) in phosphate-buffered saline (PBS). The resulting mixture was incubated at 4°C overnight. Ni-NTA (0.5 ml) was added to the reaction mixture and incubated for 20 minutes to remove sortase and unreacted VHHs. The mixture was centrifuged and the supernatant was collected and purified by size exclusion chromatography (Superdex 75- GE Life Sciences), and analyzed by SDS-PAGE and LC-MS. PEGylated VHH-X118 was generated by reacting the bioorthogonal azide group with dibenzocyclooctyne DBCO-(PEG)_20_ overnight. The end product was analyzed by SDS-PAGE to confirm efficient coupling.

### Virus Quantification

IAV was quantified by flow cytometry, using a method adapted from one previously described (25). Briefly, confluent Madin-Darby canine kidney cells were infected in triplicate with 2-fold serial dilutions of IAV WSN/33 (in DMEM, 0.2% BSA) for 1 hour. The inoculum was removed and replaced with DMEM, 0.2% (w/v) BSA for 5 hours. Cells were washed with PBS, trypsinized, and fixed with 4% formaldehyde in PBS. Cells were stained with 1 μg/ml VHH62 (anti-IAV NP)-Alexa Fluor 647 under permeabilizing conditions. Fluorescence was quantified using a BD Accuri C6 Plus. The NP-positive population was determined by comparison with an uninfected control population (**Figure S1A**). Data were processed using the FlowJo software package (TreeStar Inc). A linear regression model was applied (**Figure S1B**). The slope of the line of best fit was used to determine the percentage of infected cells, which was multiplied by the number of cells per well to yield the number of viral particles per well.

### Infection of Mice With IAV

Age-matched, 6-week old, female CD45.2 C57BL/6J mice (n≥3 in each group) were purchased from the Jackson Laboratory. Mice were anesthetized with isoflurane and infected by the intranasal route with 4 x 10^4^ infectious units of IAV WSN/33 diluted in PBS. Control mice were similarly given an equal volume of PBS intranasally. Infection was tracked by monitoring daily weight loss. Mice were euthanized with CO_2_ when weight loss exceeded 25% of initial body weight and/or animals displayed signs of severe distress, or if no weight was recovered 9 dpi.

### Immuno-PET Imaging

IAV-infected and control mice were anesthetized using isoflurane and injected *via* the retro-orbital route with approximately 25 μCi of ^89^Zr-VHH-X118-PEG_20_. PET-CT procedures have been described in detail (21, 26). Briefly, approximately 24 h post-administration of ^89^Zr-VHH-X118-PEG_20_, mice were anesthetized using 2% isoflurane in O_2_ at a flow rate of approximately 1 L/minute. Mice were imaged with a G8 PET-CT small-animal scanner (PerkinElmer). PET images were acquired over a 20 minute period, which was followed by approximately 2 minutes of CT acquisition. As a standard for absolute intensity, a fixed quantity of radioactivity was imaged using 5-fold serial dilutions of radioisotope in PCR strip tubes.

### PET Quantification

We processed and quantified PET images using VivoQuant software. The CT scan was used as a guide to generate 3D regions of interest (ROIs) to represent regions corresponding to the lungs and MLN. ROIs were drawn for each image corresponding to the absence of CT signal in the ribcage, surrounding the heart as a means of identifying pulmonary space. We used preset CT values to view the area corresponding to the lungs, and preset CT intensities (-1400, 400) were used to generate the lung ROIs. For the MLN region, ROIs were generated by creating a spherical region with a 10-pixel diameter centered around the point of highest PET intensity of MLN. An additional ROI was drawn in the quadriceps muscle of the hind leg of each mouse, avoiding bones and LNs, which was subtracted as background. Once all ROIs were generated, statistical information for each ROI containing mean PET signal, was exported and processed. We normalized signal intensities to background signal for each mouse by using the ratio between PET signal in the lungs or MLN to PET signal in the quadriceps muscle. The level of significance was determined using a Student’s t-test.

### T Cell Purification

Mice were euthanized by asphyxiation with CO_2_ and spleens were extracted by dissection. Spleens were homogenized into single-cell suspensions. Red blood cells were removed by hypotonic lysis. T cells were isolated from these splenocytes using the Dynabeads^®^ Untouched™ Mouse T Cells Kit following the suppliers’ recommended procedures. The lungs of day 9-infected CD45.2 C57BL/6J mice were extracted by dissection, and T cells were isolated using the Dynabeads^®^ Untouched™ Mouse T Cells Kit with additional incubations with anti-CD326 and anti-CD31 antibodies to deplete epithelial and endothelial cells, respectively.

### Flow Cytometry

Aside from virus quantification assays, all data were acquired on a Fortessa instrument (BD Biosciences) and analyzed using FlowJo software Cells obtained from the lungs and MLN of CD45.2 C57BL/6J mice were used for flow cytometry. Cells were resuspended in PBS (137 mM NaCl, 2.7 mM KCl, 10 mM Na_2_HPO_4_, 1.8 mM KH_2_PO_4_) with 2% (v/v) fetal bovine serum and passed through 40-μm cell strainers to obtain single-cell suspensions prior to antibody staining (30 minutes at 4°C). All antibodies were obtained from BioLegend (San Diego, CA). Student’s t-test was applied for statistical analysis.

### ELISpot Assay

96-well ELISpot plates (BD ELISPOT Mouse IFNγ ELISPOT Set, BD Biosciences, San Jose, CA) were coated with an IFNγ capture antibody (BD Biosciences) in PBS overnight at 4°C, followed by incubation with complete RPMI-1640 medium for 2 hours at room temperature (RT). Single cell suspensions from selected organs of IAV-infected and uninfected mice were prepared. Red blood cells were removed by hypotonic lysis. Quadruplicate ELISpot wells containing mononuclear cells, were supplemented with the IAV NP peptide (366-374; ASNENMETM) (2 μg/ml), to serve as a H2-D^b^-restricted epitope from the Influenza A/PR/8/34 nucleoprotein (27, 28). As a control, medium without added IAV peptide was used. ELISpot plates were incubated at 37°C for 18 hours, washed and incubated with a biotinylated IFNγ detection antibody (BD Biosciences) for 2 hours, followed by incubation with a streptavidin-horse radish peroxidase (HRP) conjugate (BD Biosciences) for 1 hour at RT. ELISpot plates were developed with 3-amino-9-ethyl-carbazole substrate (BD ELISPOT AEC Substrate Set) and dried. Spots were counted using an ImmunoSpot Analyzer.

### *Ex Vivo* T Cell Activation

Splenocytes from CD45.2 C57BL/6J mice were cultured in plates pre-coated with anti-CD3 (5 µg/ml) and anti-CD28 (1 µg/ml) antibodies in complete RPMI-1640 medium supplemented with 250 ng/ml of mouse IL-2 produced in house. Following activation for 24 hours, cells were transferred to fresh dishes without anti-CD3 and anti-CD28 in complete RPMI-1640 supplemented with 250 ng/ml of mouse IL-2, followed by two additional days of culture. Cells were washed three times with PBS and cell numbers were determined prior to transfer experiments.

### T Cell Transfer

Cells were labeled with non-PEGylated ^89^Zr-VHH-X118 in PBS at 4°C for 20 minutes with constant agitation. Cells were then washed three times with PBS to remove unbound ^89^Zr-VHH-X118. Labeled cells (6 x 10^6^) were then transferred into the retro-orbital plexus of each CD45.1 C57BL/6J mouse. Images were acquired as previously described 1 and 24 h post-T cell transfer.

### FACS

T cells purified from lungs of day 9-infected CD45.2 BL/6J mice were stained with FITC anti-mouse CD8 and Alexa700 anti-mouse CD45.2 antibodies (BioLegend). The CD8^+^CD45.2^+^ T cell population was sorted on a BD FACSAria III sorter.

### Ethics Statement

All animal protocols were conducted in accordance with the Guide for the Care and Use of Laboratory Animals of the National Institutes of Health. All animals were maintained according to the guidelines of the Animal Resources Children’s Hospital. These studies were approved by the Boston Children’s Hospital Institutional Animal Care and Use Committee (protocol #16-12-3328). All infections and PET imaging procedures were performed under isoflurane anesthesia and all efforts were made to minimize suffering.

## Results

### VHH Construct Design for Immuno-PET Imaging of CD8^+^ T Cells

**Figure 1:**
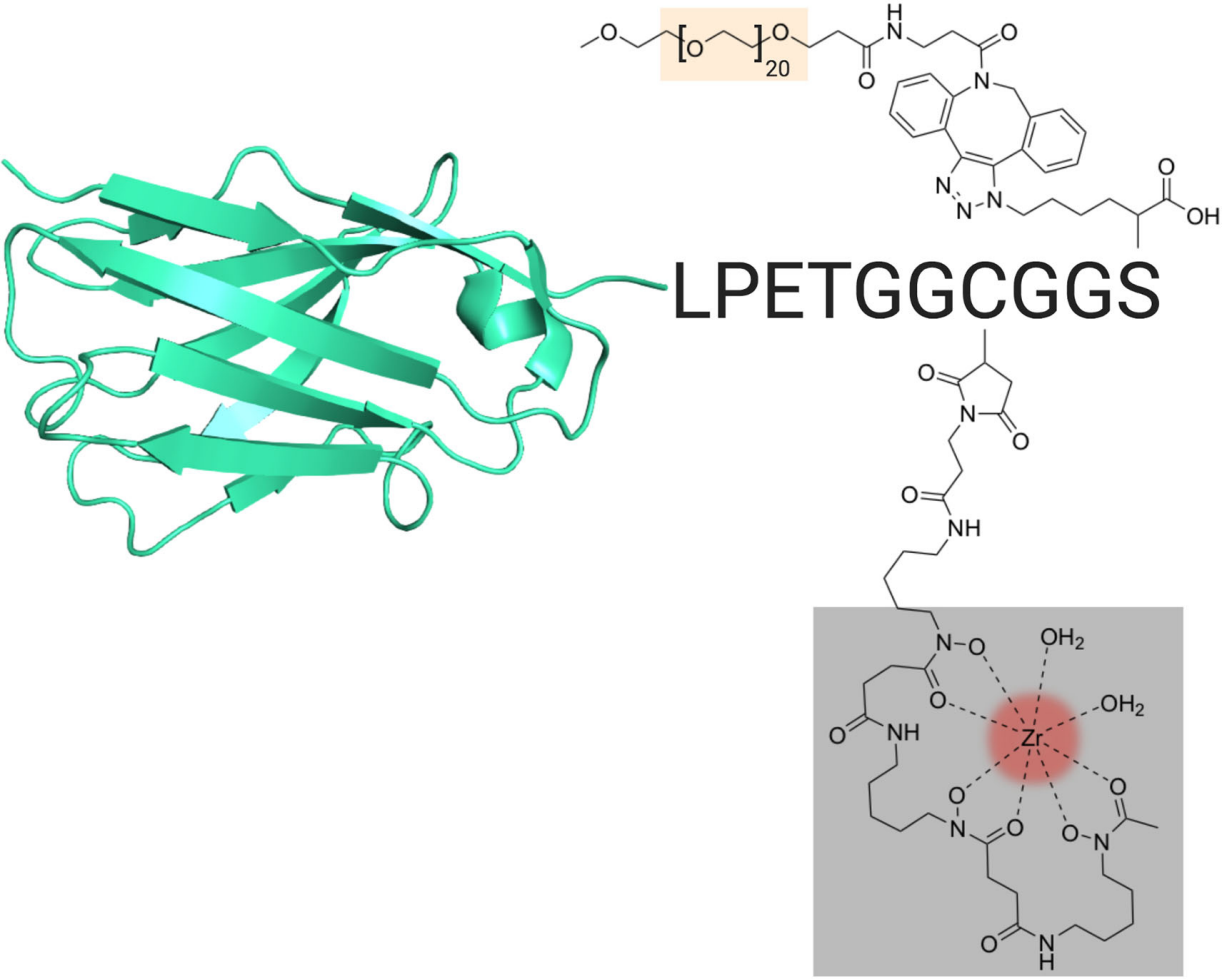
VHH Construct and Design. Schematic of the VHH-X118 construct used to track CD8^+^ T cells *in vivo*. A representative VHH structure (PDB: 3OGO) is shown covalently bound to the peptide probe LPETGGCGGS. Maleimido-DFO (grey), which was covalently linked to the peptide probe at the cysteine thiol, is shown chelating ^89^Zr (red). A terminal, azide-substituted lysine was covalently modified with a PEG_20_ substrate (peach) for improved circulation.

In order to follow CD8^+^ T cells noninvasively over the course of IAV infection, we used a VHH specific for murine CD8α, VHH-X118 (21). We produced two versions of ^89^Zr-labeled VHH-X118, both made *via* modification of the VHH’s C-terminal sortase recognition motif (LPETG). This modification allows the use of a peptide containing an N-terminal GGGC sequence equipped with a radiometal chelator to serve as a nucleophile in a sortase A-mediated transpeptidation reaction (29). By incorporation of maleimido-desferrioxamine (DFO), we installed ^89^Zr for PET imaging. We also generated a PEGylated version of ^89^Zr-labeled VHH-X118 by using an azide-substituted lysine in the course of synthesis of the nucleophile to incorporate polyethylene glycol (PEG) moieties for *in vivo* injections, effectively reducing non-specific retention in the kidneys (**Figure 1**) (21). The agent used for *ex vivo* labeling of CD8^+^ T cells prior to use in transfer experiments, as described below, did not include a PEG moiety.

### CD8^+^ T Cells Transiently Accumulate in the Lungs and Mediastinal Lymph Nodes (MLN) of IAV-Infected Mice, Correlating With Morbidity

**Figure 2:**
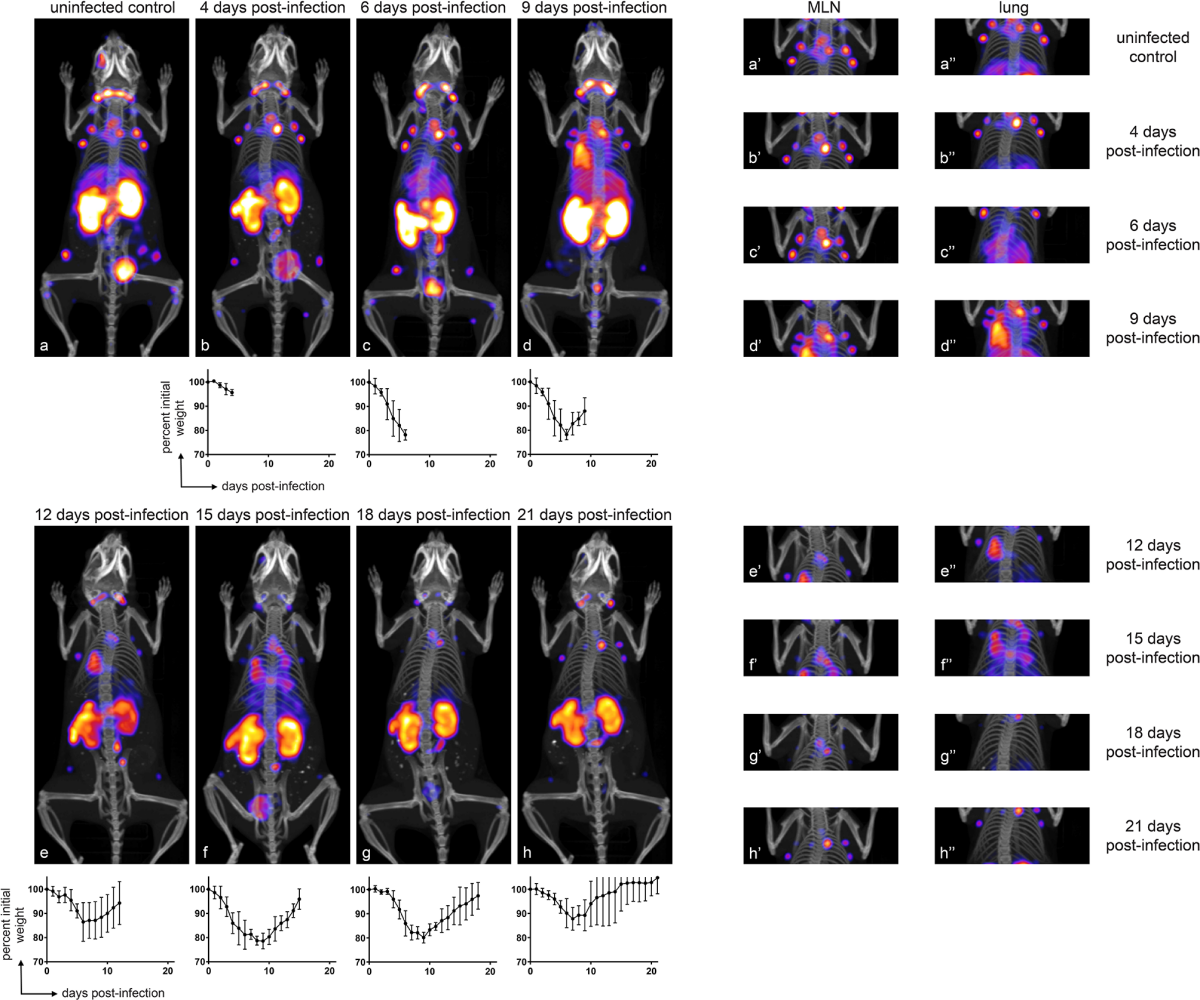
CD8^+^ T Cells Transiently Accumulate in the Lungs and MLN of IAV-Infected Mice, Correlating with Morbidity. Representative (n ≥ 3) immuno-PET images of IAV WSN/33-infected mice (facing down) injected with ^89^Zr-labeled VHH-X118-PEG_20_ on the indicated dpi. Graphs below immuno-PET images show percent initial weight for each day over the course of IAV infection for the cohort of animals. Insets to the right depict focused images of the MLN and lungs at the times indicated. Lower case letters link inset images to their corresponding full-body image, and single and double apostrophes indicate MLN and lung insets, respectively.

To establish the whole-body distribution of CD8^+^ T cells during IAV infection, we acquired CD8^+^ PET images using ^89^Zr-labeled VHH-X118, and in parallel, tracked weight loss after intranasal inoculation of CD45.2 C57BL/6J mice (n≥3 for each group) with a sub-lethal dose (4 x 10^4^ infectious units) of influenza A/WSN/33 virus (H1N1). In uninfected mice, CD8^+^ T cells were distributed in the cervical, axillary and brachial (lung draining), mediastinal (lung draining), popliteal, renal, iliac, and inguinal lymph nodes, as well as in the spleen, consistent with previous observations (21). PET signals in the organs of elimination (kidneys, liver and bladder) and the site of injection (retro-orbital plexus) are non-specific and common occurrences when using nanobodies as imaging agents (21, 30, 31). During the first week of IAV infection, mice experienced weight loss, paralleled by increases in PET signal in the mediastinal lymph node (MLN) and lungs (**Figure 2**). Attribution of PET signals to particular anatomical structures was confirmed by imaging dissected organs, including MLN, lungs, heart, and thymus (**Figure S2**). At 4 days post-infection (dpi), mice showed a striking increase in CD8^+^ T cells in the area corresponding to the MLN and in the draining lymph nodes, as inferred from the PET signal. At the peak of infection (6 dpi) as determined by weight loss, a diffuse pattern of CD8 signal was present in the lungs, likely caused by CD8^+^ T cell migration from secondary lymphoid organs towards particular foci of infection. This signal became more concentrated and localized in specific regions of the lungs over the course of the infection, starting at 9 dpi. The PET signal decreased around 18 dpi, when mice regained weight in the recovery phase, and finally disappeared from the lungs by 21 dpi.

### Immuno-PET Shows Accumulation of CD8^+^ T Cells in the Lungs and MLN of IAV-Infected Mice

**Figure 3:**
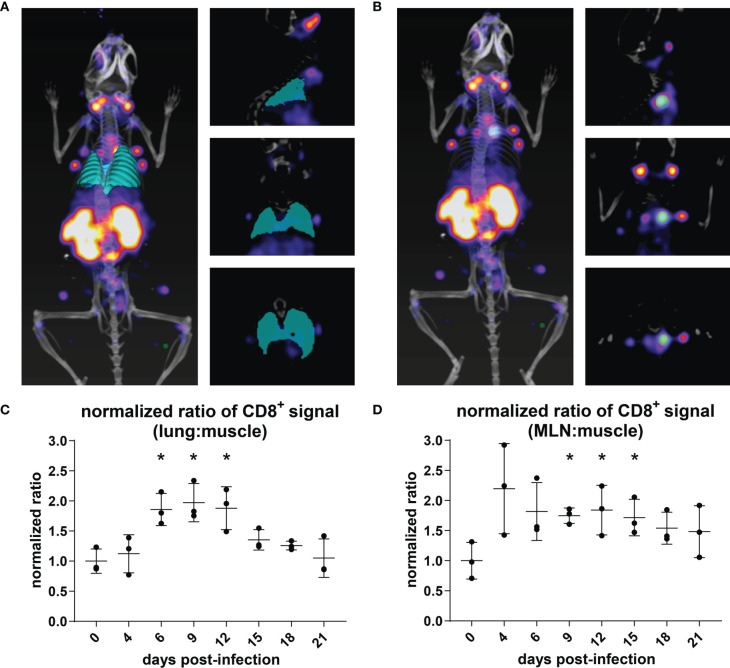
Quantification of CD8^+^ T Cell Immuno-PET Signal in the Lungs and MLN/Thymus. **(A, B)** The volume of the lungs (cyan) was determined using the VivoQuant 3D ROI tool in each mouse based on CT signal (white/grey) **(A)**. The volume of the MLN (cyan) was defined based on PET intensity **(B)**. In each mouse, PET intensity was normalized to background signal as defined by a ROI in the quadriceps muscle (green). Mice are shown facing down as 3D renderings (left) and in slices: frontal (top right), sagittal (middle right), and transverse (bottom right) ROI. **(C, D)** PET intensity from the lung ROI **(C)** for each day was normalized to the PET intensity of day 0, uninfected mice (n = 3). Student’s t-tests were used to compare infected mice at each time point to uninfected mice. *P < 0.05.

In order to determine whether the PET signals observed in the lungs and MLN of mice over the course of infection constituted measurable significant increases, we quantified the PET signal from the animals in **Figure 2** using VivoQuant software. Specifically, we generated 3D regions of interest (ROIs) representing the lungs (**Figure 3A**; cyan), MLN (**Figure 3B**; cyan), and quadriceps muscle (control; green) to quantify the PET signal within the volume of the ROI. Mean intensity was calculated by dividing the PET signal by the volume of the ROI. This value was further normalized within each mouse to the intensity of the quadriceps muscle, in order to account for possible variations in multiple experiments and across mice. CD8 signal intensity in the lungs increased during infection and remained elevated from days 6 through 12 of IAV infection. Lung signal decreased after day 12, as mice recovered from IAV infection. These results correlate with morbidity, as mice reached the peak of their weight loss in this interval (**Figure 2**, inset graphs). Similarly, we observed a trend towards an increase in CD8 signal intensity in the MLN beginning at 4 dpi, which reached significance (P<0.05) between days 9 and 15 of the infection, before eventually decreasing during the recovery phase of the infection (**Figures 3C, D**). An increase in CD8 signal intensity should be detectable in the MLN of infected mice prior to its appearance in the lungs, as T cells are activated in lymph nodes prior to migrating to the lungs in response to chemokines (32).

### T Cells in Day 9-Infected Lungs Are Predominantly CD8^+^ T_EFF_/T_EM_ Cells

**Figure 4:**
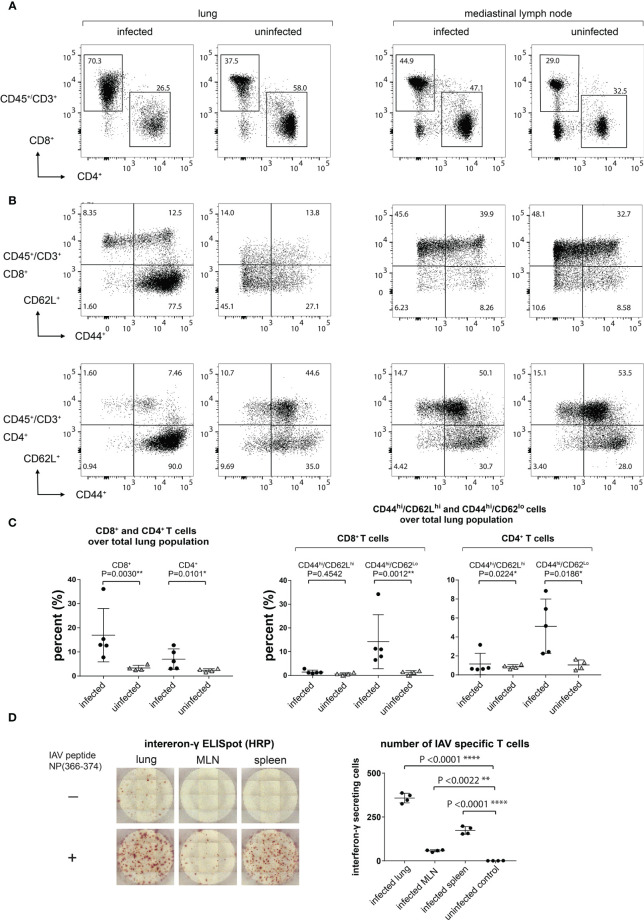
Lung-Infiltrating Cells are Predominantly CD8^+^ T_EFF_/T_EM_ Cells, a Significant Amount of Which are IAV Specific. **(A)** The infiltrating T cells were gated on CD45^+^CD3^+^ and the ratio of CD4^+^ and CD8^+^ was determined. **(B)** CD8^+^ and CD4^+^ T_EFF_/T_EM_ (CD44^hi^CD62L^lo^) and T_CM_ (CD44^hi^CD62L^hi^) were gated on CD45^+^CD3^+^CD4^+^CD44^+^CD62L^-^ and CD45^+^CD3^+^CD8^+^CD44^+^CD62L^+^. **(C)** Statistical analysis of data from **(A, B)**. **(D)** IAV-specific T cell responses in lungs, MLN, and spleen as determined by an ELISpot assay. Representative ELISpot wells are shown in the left panel and statistical analyses of lungs, MLN, and spleen IAV-specific T cells are shown in the right panel.

To characterize the population of infiltrating T cells at 9 dpi and uninfected mice, we performed flow cytometry on cells isolated from the lungs and MLN. The ratio of infiltrating CD4^+^ versus CD8^+^ T cells was determined by gating on the CD3^+^CD45^+^ population (**Figure 4A**). The number of infiltrating lymphocytes in the lungs of day 9-infected mice was significantly increased (**Figure 4C**), in agreement with the observed increase in PET signal. To better identify the T cell subset present in the infiltrating population and to establish the percentage of CD44^+^CD62L^+^ [central memory T cells (T_CM_)] and CD44^+^CD62L^-^ [effector T cells (T_EFF_/T_EM_)] cells, we gated on either the CD8^+^ or CD4^+^ T cell population (**Figure 4B**). The majority of infiltrating CD8^+^ and CD4^+^ T cells in the lungs were CD44^+^CD62L^-^ (T_EFF_/T_EM_). No significant increase in CD8^+^CD44^+^CD62L^+^(T_CM_) was detected in the lungs of the infected animals (**Figure 4C**).

### The Lung-Infiltrating Population Contains IAV-Specific T Cells

We next confirmed that IAV-specific T cell responses could be detected in the lungs, MLN, and spleen by using a peptide corresponding to the immunodominant H-2D^b^-restricted IAV NP epitope, ASNENMETM, in an IFNγ ELISpot assay. As expected, we saw an increase in the number of IAV-specific CD8^+^ T cells in the lungs, MLN, and spleen in infected mice (**Figure 4D**). We detected low levels of IFNγ-producing cells in the media-only controls in the lungs from infected animals, presumably due to the persistence of antigen and/or previously activated IFNγ-producing T cells.

### Distribution of Transferred CD8^+^ T Cells Relies on the Mechanism of T Cell Activation and the Local Inflammatory Environment

**Figure 5:**
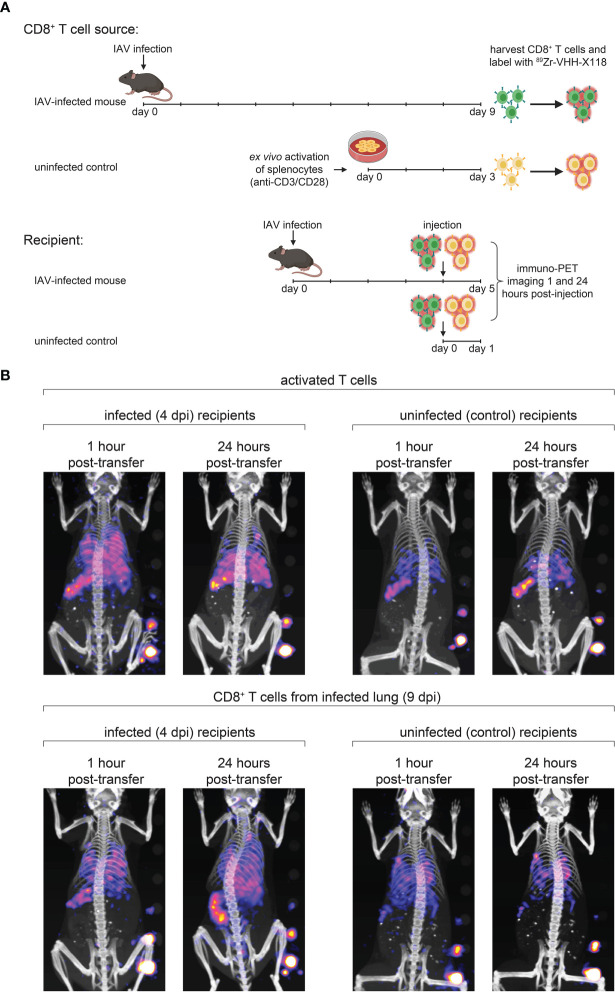
Distribution of Transferred CD8^+^ T Cells Relies on the Site of T Cell Maturation and Local Inflammatory Environment. **(A)** Schematic depicting CD8^+^ T cell transfer experiments. Donor mice were infected with IAV WSN/33 and CD8^+^ T cells were harvested from the lungs at 9 dpi. Harvested CD8^+^ T cells were *ex vivo* labeled with ^89^Zr-VHH-X118 and injected into day 4-infected or uninfected mice. As a control, CD8^+^ T cells were purified from naïve splenocytes of uninfected mice, *ex vivo* activated, and injected into day 4-infected and uninfected mice. Mice were immuno-PET imaged 1 hour and 24 hours post-transfer. **(B)** Day 4-infected or uninfected recipient mice, shown face down, were immuno-PET imaged at the indicated times post-transfer of ^89^Zr-labeled T cells from the indicated donor source. A standard with set amounts of radioactivity in 2-fold dilutions starting at 0.4 μCi is on the lower right side of each image. Data are representative of 2 experiments.

Transfer of virus-specific CD8^+^ T cells can clear IAV in a B cell-deficient mouse model (33). In such settings, the tissue distribution of donor CD8^+^ T cells remains unclear. Does the inflamed environment of the lungs provide signals that direct and retain T cells? Do T cells instructed to deal with a pulmonary insult display lung homing properties, independent of whether the lungs are infected or not? In any case, little is known about the *in vivo* distribution of T cells immediately after transfer into recipients. We approached these questions by transfer of ^89^Zr anti-CD8α-labeled CD8^+^ T cells to trace their distribution in the recipients at 1 hour and 24 hours post-transfer, using whole-body immuno-PET imaging. T cells were harvested and purified from the lungs of IAV-infected mice 9 dpi, with additional negative selection steps to remove epithelial and endothelial cells, followed by cytofluorimetry to assess purity of the T cell population to be transferred. We likewise harvested splenocytes from uninfected control mice and purified CD8^+^ T cells from them. These naïve T cells were then activated on plate-bound anti-CD3 and anti-CD28, in medium supplemented with IL-2 (34). CD8^+^ T cells from day 9-infected mice or activated CD8^+^ T cells from control mice were labeled in suspension with ^89^Zr-VHH-X118. Labeled cells were then transferred into either IAV-infected (4 dpi) or uninfected control mice *via* retro-orbital injection. PET images were acquired at 1 hour and 24 hours post-transfer (**Figure 5A**).

Upon transfer into day 4-infected recipient mice, *ex vivo* activated, control CD8^+^ T cells initially localized to the lungs and spleen (**Figure 5B**, top panels). After 24 hours, the signal corresponding to CD8^+^ T cells decreased in the lungs. Activated CD8^+^ T cells generated from control splenocytes transferred into naïve mice localized mostly to the spleen and remained there after 24 hours. CD8^+^ T cells from day 9-infected mice initially migrated to the lungs and spleen when transferred into day 4-infected and uninfected recipients, imaged 1 hour post-transfer (**Figure 5B**, bottom panels). After 24 hours, a signal corresponding to these CD8^+^ T cells remained detectable in the lungs of day 4-infected mice and appeared to increase in intensity in the spleen and MLN. Uninfected mice also retained a signal corresponding to CD8^+^ T cells from infected mice in their lungs 24 hours post-transfer, but to a lesser extent, probably due to the lack of an inflammatory environment. CD8^+^ T cells harvested from day 9-infected mice did not initially populate the spleens of uninfected mice in large numbers, nor did they appear to migrate there over the 24 hour observation period.

## Discussion

We determined the whole-body distribution of CD8^+^ T cells in a living mouse in the course of infection with IAV. An increase in CD8^+^ T cells was first detectable in lymph nodes, specifically the MLN, where CD8^+^ T cells are activated by antigen presenting cells that present IAV-specific antigens (35). CD8^+^ T cells then migrate to the lungs in response to chemotactic cues, where they can be detected diffusely, but in significant numbers at 6 dpi, when mice experience peak weight loss. At 9 dpi, CD8^+^ T cells showed more discrete sites of accumulation in the infected lungs. They remained there in elevated numbers until 12 dpi. CD8^+^ T cells then left the lungs as mice recovered. The CD8^+^ T cell signal remained elevated in the MLN throughout the infection, followed by a gradual decrease in signal strength as the infection waned.

Following viral infection, most pathogen-specific T cells undergo a process of contraction by apoptosis. A small fraction of the remaining antigen-specific T cells differentiate into memory cells (9). Effector memory T cells (CD8^+^CD44^+^CD62L^-^) recirculate in the blood and non-lymphoid tissues. They protect the host against re-infection by their ability to kill cells infected with influenza virus and by the production of inflammatory cytokines at the site of infection (36). Central memory T cells (CD8^+^CD44^+^CD62L^+^) take up residence in secondary lymphoid organs. They proliferate, differentiate, and migrate to new sites of infection (9). At 9 dpi with IAV, most CD8^+^ T cells in the lungs are effector memory T cells.

**Figure 6:**
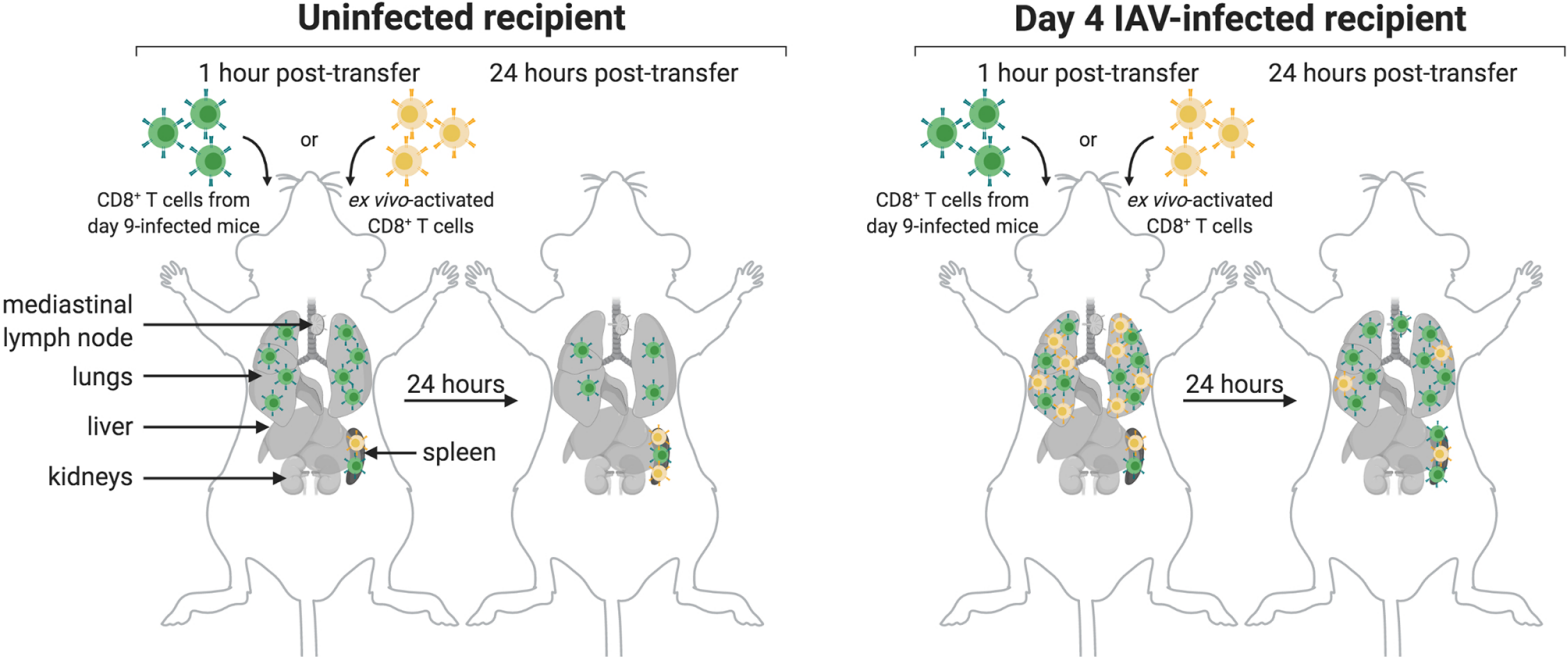
Model of CD8^+^ T Cell Transfer to Infected and Uninfected Mice. CD8^+^ T cells transferred from day 9-infected mice (green) initially localize to the lungs and spleen of uninfected (left) and IAV-infected (right) mice. After 24 hours, these cells are retained in the lungs and spleen of both uninfected and infected mice, though retention is not as strong in the lungs of uninfected mice. Additionally, a subset of these cells migrates to the MLN of infected mice after 24 hours. Transferred *ex vivo* activated CD8^+^ T cells (yellow) initially localize to the spleen of both uninfected and infected mice, and in both cases remain there after 24 hours. While these cells do not localize to the lungs of uninfected mice, they do transiently populate the lungs of infected mice; however, these cells are not retained in the lungs after 24 hours.

As summarized in **Figure 6**, CD8^+^ T cells obtained from IAV-infected mice (9 dpi), upon transfer, initially populate the lungs of both infected and uninfected mice. A CD8 signal remains detectable there after 24 hours. Lymphocyte function-associated antigen 1 (LFA-1) not only directs migration but also mediates retention of effector CD8^+^ T cells in the lungs. LFA-1 interacts with intercellular adhesion molecules (ICAMs) on endothelial cells to allow leukocyte migration across the endothelium to sites of inflammation (37). Normal, healthy lung tissue supports retention of activated CD8^+^ T cells. Indeed, ICAM-1, an LFA-1 ligand, is expressed in healthy lung tissue (38). In contrast, T cells activated *ex vivo* with anti-CD3 and anti-CD28 more transiently localized to the lungs of IAV-infected recipients, and did not populate the lungs of uninfected recipients in a manner that was detectable by immuno-PET imaging. This suggests that the inflamed status of the lungs provides signals, probably in the form of both soluble mediators and surface molecules, that allow retention of activated T cells, regardless of their specificity, whereas healthy lungs do not provide such cues. *Ex vivo* activated CD8^+^ T cells initially populated the lungs of IAV-infected mice, presumably due to the pro-inflammatory signals resulting from infection. Signal is still detectable in the lungs 24 hours post-transfer, albeit of lesser intensity. We hypothesize that the absence of IAV antigens from control mice and the corresponding lack of continued engagement of the TCRs on transferred *ex vivo* activated T cells likely contribute to this phenotype. Further, the gene expression profiles of *ex vivo* activated and *in vivo* activated IAV-specific CD8^+^ T cells likely differ, which may contribute to their ability to interact with lung-expressed retention ligands. It is also possible that IAV-specific CD8^+^ T cells die upon transfer to uninfected recipients, which could explain why they appear to be retained in the lungs; however, it is unclear why antigen-specific CD8^+^ T cells would die, while activated, non-specific T cells would retain the capacity to migrate post-transfer. These are subjects of future investigation.

In this study we show that ^89^Zr-labeled single-domain antibody fragments can track the CD8^+^ T cell response to IAV infection noninvasively in a living mouse. Nanobodies have several advantages for use in PET imaging when compared to intact Ig or Fab fragments. The small size of a nanobody (~12–16 kDa) enables access to epitopes not available for binding to conventional Ig, provides improved tissue penetration, high stability, and rapid renal clearance from the body of unbound nanobodies (39–42). Further, nanobodies can be produced in high yield and in active form in bacteria (43). A possible drawback of nanobodies for *in vivo* use is their immunogenicity; while nanobodies are typically poorly immunogenic *in vivo*, this is not always the case. Even then, immunogenicity can often be modulated by modification of the framework regions of the construct (39, 40, 44, 45).

The platform used in this study is highly amenable to adaptation. Addition of a sortase recognition motif to the C-terminus of a nanobody enables rapid and efficient covalent modification of the nanobody. We used sortase to append a radiometal chelator to the nanobody of interest. Modification of this strategy is simple owing to the commercial availability of many different chelators with various functionalized handles. The sortase reaction also enabled us to append PEG_20_ to the nanobody of interest to increase circulatory half-life and decrease renal retention of the construct; PEGylation of a construct is not always required, and the size of the PEG moiety can be adjusted according to the needs of the experiment (43). The choice of available PET isotopes and their characteristic half-lives (^68^Ga: ~60 min; ^18^F: ~110 min; ^64^Cu:~12 hrs; ^89^Zr: ~3.3 days) sets differing observation windows, which can be matched to the immunological parameters of interest by the choice of a VHH of appropriate specificity.

We propose that immuno-PET can be used to study the immune response to any infection that induces CD8^+^ T cells The signature advantage of this method is its noninvasiveness. More conventional methods require sacrificing the animals to enable organ retrieval by dissection. Additional VHHs can, of course, be developed against antigens from other animal models and can be used to track various immune cells. Because of this, we propose that immuno-PET can be utilized to monitor immune responses to vaccines and to garner a better understanding of their mechanisms of action.

## Data Availability Statement

The raw data supporting the conclusions of this article will be made available by the authors, without undue reservation.

## Ethics Statement

The animal study was reviewed and approved by Boston Children’s Hospital Institutional Animal Care and Use Committee.

## Author Contributions

PR, ZL, and HP contributed to conception and design of the study. Experiments were performed by PR and ZL with experimental assistance provided by NP, YX, AW, DB, SK, and VV. PR and ZL analyzed the data and performed statistical analyses with insight from NP and AW. PR, ZL, and HP drafted the manuscript with supportive feedback from NP, YX, AW, DB, SK, and VV. All authors contributed to the article and approved the submitted version.

## Conflict of Interest

The authors declare that the research was conducted in the absence of any commercial or financial relationships that could be construed as a potential conflict of interest.

## Publisher’s Note

All claims expressed in this article are solely those of the authors and do not necessarily represent those of their affiliated organizations, or those of the publisher, the editors and the reviewers. Any product that may be evaluated in this article, or claim that may be made by its manufacturer, is not guaranteed or endorsed by the publisher.
