# Supplementary material for: Noninvasive Immuno-PET Imaging of CD8+ T Cell Behavior in Influenza A Virus-Infected Mice
Source: Front Immunol. 2021 Nov 3;12:777739. doi: 10.3389/fimmu.2021.777739 (PMC8595544; doi:10.3389/fimmu.2021.777739)
Supplement: Supplementary file 1 [file DataSheet_1.docx]

**
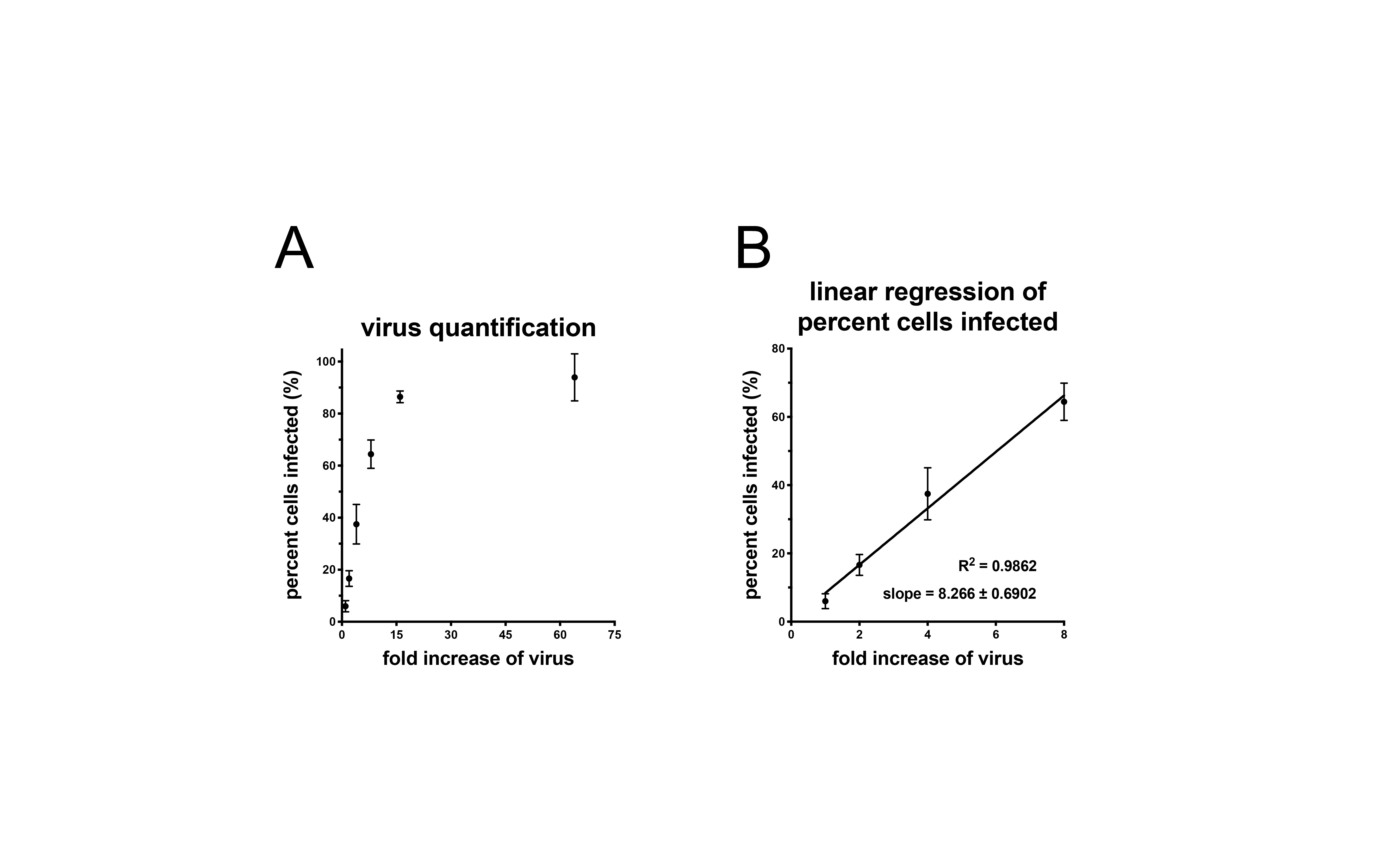
**

**Figure S1. Virus Quantification for *In Vivo* Infections.** (*A*) Madin-Darby canine kidney cells were infected in triplicate with increasing dilutions of IAV WSN/33 for 1 hour. Inoculum was removed and cells were incubated for an additional 5 hours. Cells were fixed and stained with IAV NP-specific VHH62-Alexa Fluor 647 under permeabilizing conditions. Fluorescence was quantified by flow cytometry. (*B*) A linear regression model was applied to the linear portion of the data in (*A*) and the slope of the line of best fit was used to determine the percentage of infected cells. The number of infectious viral particles per well, and thus the virus concentration, was then back-calculated from the slope and the number of cells per well.


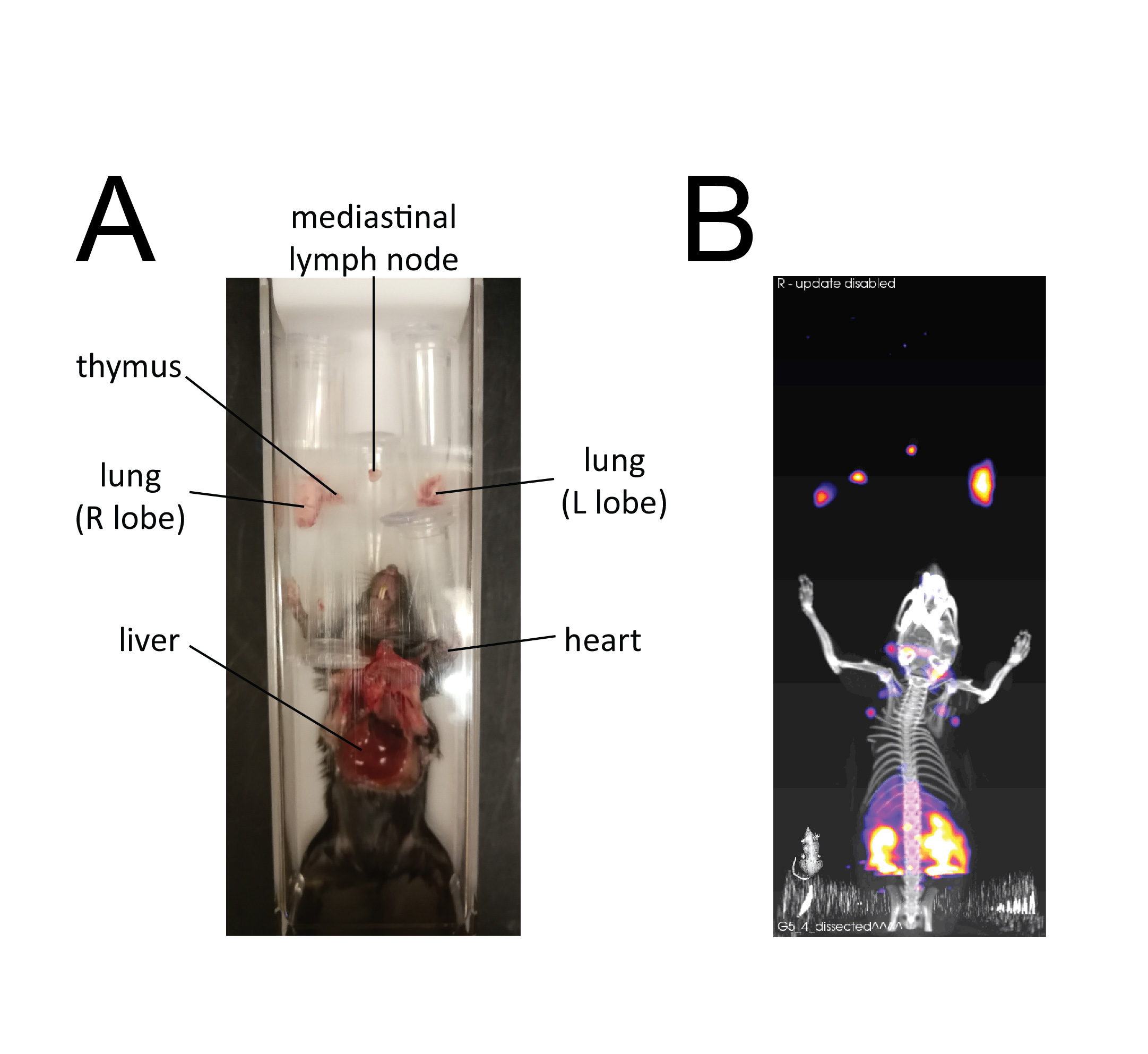


**Figure S2. Confirmation of Correct Identification of Organs by Immuno-PET Imaging of Dissected Organs.** (*A*) A representative sacrificed and dissected IAV WSN/33-infected mouse, which had previously been used for immuno-PET imaging with ^89^Zr-VHH-X118-PEG_20_, is shown face up. (*B*) PET signal confirms the identification of various organs, including the thymus, lungs, liver and mediastinal lymph node. Signal is notably not detected in the heart.
